# Supplementary material for: Bayesian refinement of protein structures and ensembles against SAXS data using molecular dynamics
Source: PLoS Comput Biol. 2017 Oct 18;13(10):e1005800. doi: 10.1371/journal.pcbi.1005800 (PMC5662244; doi:10.1371/journal.pcbi.1005800)
Supplement: S1 Archive — (BZ2) [file pcbi.1005800.s011.tar.bz2 › gromacs_4.62_bayesian_saxs_refi/share/html/online/methanol.html]

Getting started - Methanol


| Getting started - Methanol Main Table of Contents |

**VERSION 4.6  
Sat 19 Jan 2013**

---

## Methanol

Now you are going to simulate 216 molecules of methanol
in a cubic box. In this example the GROMACS
software team already generated most of the neccesary input
files. The files needed in this example are:

- Initial structure of a box of 216 methanol molecules (.gro)
- Topology file of methanol (.top)
- Molecular Dynamics parameter file (.mdp)

Change your directory to tutor/methanol :
  
  

|  |  |  |
| --- | --- | --- |
|  | cd tutor/methanol  \| |

  
Let's first have a look at the coordinate file:
  
  

|  |  |  |
| --- | --- | --- |
|  | more conf.gro   \| |

  
Or to view the methanol box graphically:
  
  

|  |  |  |
| --- | --- | --- |
|  | rasmol methanol.pdb   \| |

  
The Carbon group (CH3) is treated as one particle. In the file it
is called Me1 (methyl 1) and rasmol does not recognize it as carbon (and hence
colors it purple).

Since all the neccesary files are available, we are going to
preprocess the input files to create a run input
(.tpr) file.
This run input file is the only input file for the
MD-program mdrun.
  
  

|  |  |  |
| --- | --- | --- |
|  | grompp -v   \| |

  
Now it's time to start the simulation of 20 picoseconds.
  
  

|  |  |  |
| --- | --- | --- |
|  | mdrun -v   \| |

  
After the MD simulation is finished, it is possible to view the
trajectory with the ngmx program:
  
  

|  |  |  |
| --- | --- | --- |
|  | ngmx   \| |

  

When the program starts, you must select a group of atoms to view. In
our case that will be "MeOH" (for methanol) or "System", which is the
same for a box of methanol as we have. Select one and click OK. Then
select Display->Animate from the menu. Use the buttons to see your
methanol moving (note: "Play" steps one frame forward; "Fast Forward"
plays; "Rewind" skips back to the beginning of the trajectory).

---

## Analysis of the simulation

1. Calculate a radial distribution function of the oxygen atoms
   around oxygen atoms. The index file index.ndx now contains two groups, one with
   the methyl atoms and one with the oxygen atoms.
     
     

   |  |  |  |
   | --- | --- | --- |
   |  | g\_rdf -n index -o rdf-oo.xvg   \| |

     
   The program will ask you for how many groups you want the calculate
   the RDF, answer 1 (and select oxygen and oxygen). Now,
   view the output graph.
     
     

   |  |  |  |
   | --- | --- | --- |
   |  | xmgrace rdf-oo.xvg   \| |

     
   Which shows you the radial distribution function for oxygen-oxygen in
   Methanol. Now do the same thing using the methyl group as reference and
   as target (and use e.g. rdf-mm.xvg as output file name). View all
   the graphs together:
     
     

   |  |  |  |
   | --- | --- | --- |
   |  | xmgrace rdf-oo.xvg rdf-mm.xvg ../water/rdf.xvg -legend load   \| |

     
   The xmgrace program will display three different graphs.
   Compare the resulting graphs. Explain the differences and the
   similarities.
2. We can also do a direct analysis of the number of hydrogen bonds in
   methanol, based on O-O distance and O-H ... O angle.
     
     

   |  |  |  |
   | --- | --- | --- |
   |  | g\_hbond   \| |

     
   Select twice 0, when asked. Check the output with
     
     

   |  |  |  |
   | --- | --- | --- |
   |  | xmgrace hbnum.xvg   \| |

     
   What is the number of hydrogen bonds per methanol
   molecule? What is the maximum possible number? Compare the results to
   those from water
3. As a further test of the simulation we will compute the self
   diffusion constant of Methanol, again from the **Mean
   square displacement** (MSD) function.
     
     

   |  |  |  |
   | --- | --- | --- |
   |  | g\_msd -n index   \| |

     
   (Select Me1, or O2). View the output
     
     

   |  |  |  |
   | --- | --- | --- |
   |  | xmgrace msd.xvg   \| |

     
   Check that the graph is roughly linear. The g\_msd program also
   computes the diffusion constant D for you.
   Compare the result to water. Is it as you would expect?

---


### Go to the next step


---
